# Influence of precursor synthesis temperature and hydrothermal alkaline medium on agglomeration and crystal phase of barium titanate nanoparticles

**DOI:** 10.55730/1300-0527.3792

**Published:** 2026-03-26

**Authors:** Murat ÖZEN

**Affiliations:** Department of Chemistry, Faculty of Engineering and Natural Sciences, Bursa Technical University, Bursa, Turkiye

**Keywords:** Barium titanate, agglomeration, hydrothermal synthesis, peroxo-hydroxide precursor, perovskite, morphology

## Abstract

Phase-pure, cube-like barium titanate (BaTiO_3_) nanoparticles were synthesized via hydrothermal treatment of a single-source barium-titanium peroxo-hydroxide precursor. Precursor solutions prepared at room temperature, 0 °C, and −10 °C with various storage times exhibited Ba/Ti molar ratios ranging from 0.66 to 1.05 as determined by X-ray fluorescence. Cooling the precursor solution to −10 °C effectively suppressed the polymerization of Ti-O-Ti, yielding near-ideal stoichiometry and finer precursor particles. Subsequent hydrothermal processing in NH_4_OH, KOH, and NaOH (0.5–10 M, 100–200 °C) revealed the strong dependence of agglomeration and morphology on the alkaline medium. While NH_4_OH and KOH resulted in severe agglomeration and the formation of micrometer-scale conglomerates, hydrothermal treatment in 10 M NaOH at 200 °C produced well-faceted, weakly agglomerated cubic BaTiO_3_ particles with edge lengths of approximately 200–500 nm. The particle-size distribution was further tuned by reducing the solution volume from 60 to 10 mL and increasing the total Ba + Ti concentration from 0.05 to 0.30 M, leading to sharper cubic morphologies and bimodal size distributions at higher concentrations. X-ray diffraction combined with Rietveld refinement confirmed phase-pure BaTiO_3_ and demonstrated that the cooling rate governed the cubic-to-tetragonal phase transition through size- and strain-mediated stabilization. Fourier-transform infrared spectroscopy, Fourier-transform Raman spectroscopy, and thermogravimetric-differential thermal analysis verified characteristic titanate bonding and BaCO_3_ contamination (~20 wt.%) at high feedstock concentration. The combination of low-temperature precursor synthesis and NaOH-mediated hydrothermal processing provides a scalable strategy for preparing morphology-controlled BaTiO_3_ nanoparticles with potential relevance for electronic, electrooptic, and bioimaging applications.

## 1. Introduction

The bulk properties of any material change significantly as the crystal size decreases from the macroscopic to the molecular scale [1]. Nanoscale materials are of vital importance to the electronic and electrooptical industries owing to their extraordinary mechanical, electrical, optical, and magnetic properties. Perovskite-structured metal oxides, such as barium titanate (BaTiO_3_), are widely employed in various electronic applications because of their unique ferroelectric, pyroelectric, and piezoelectric behaviors [2,3]. BaTiO_3_, one of the most extensively studied dielectric materials, has attracted considerable attention for potential applications in nanoscale devices.

A major application of nano-BaTiO_3_ lies in medical imaging techniques, such as second harmonic generation (SHG). The nanomaterial emits radiation at half the incident wavelength when irradiated with a laser of a given wavelength, enabling molecular-level imaging of live cells and tissues without surgical intervention [4]. Tetragonal BaTiO_3_ (t-BaTiO_3_) exhibits strong SHG signals under moderate to weak excitation intensities. By labeling specific genes or proteins with nano-t-BaTiO_3_, researchers can monitor the functionality and interactions of the tagged biomolecules in real time [5–7].

Among various synthesis techniques, the hydrothermal method is one of the most important approaches for producing nano-BaTiO_3_ [8]. However, reports in the literature often describe the severe agglomeration of the resulting nanoparticles. Depending on the extent of the agglomeration, particles may conglomerate to the micrometer range, thereby forfeiting their true nanoscale characteristics. In this study, the term “nanoparticle” is used to describe BaTiO_3_ particles whose properties are governed by nanoscale-derived effects, including size-dependent structural behavior, single-crystalline character, and functional responses distinct from bulk BaTiO_3_ [9–14]. Although the materials synthesized fall within the submicron size regime, this terminology is retained to reflect their nanoscale-related physicochemical and functional characteristics.

Hydrothermal synthesis has emerged as a versatile low-temperature route for producing BaTiO_3_ powders with controlled crystallinity, morphology, and particle size, avoiding the severe agglomeration typically associated with solid-state processing. Using soluble or semisoluble titanium precursors, dissolution–precipitation mechanisms dominate, yielding near-spherical or weakly faceted nanoparticles with narrow size distributions in the range of 60–200 nm and high crystallinity, but often with surface-adsorbed hydroxyl species that promote soft agglomeration [15–17]. The choice of precursor chemistry and mineralizer strongly influences growth kinetics and aggregation behavior, with TiO_2_-based in situ transformation routes generally producing larger hollow or bowl-like particles and broader size distributions due to diffusion-limited growth [15,17]. Agglomeration control has been widely addressed with organic additives, such as polyvinyl alcohol (PVA) or polyvinylpyrrolidone (PVP), which adsorb on particle surfaces via hydrogen bonding. These organic additives suppress hard agglomeration and enable well-dispersed, single-crystalline nanoparticles down to ~70 nm, although residual organics may affect downstream densification [18,19]. Highly monodisperse cube-like BaTiO_3_ nanocrystals with sizes below 30 nm have been achieved by surfactant-assisted hydrothermal routes employing oleic acid or amines, where selective stabilization of the facet governs morphology and limits aggregation [20,21]. These studies demonstrate that hydrothermal BaTiO_3_ synthesis offers fine control over particle size and dispersion, while agglomeration remains closely linked to surface chemistry, precursor solubility, and postsynthesis hydroxyl or organic residues.

The novel aspect of the present study is the use of a high-concentration mineral base without the requirement of calcination, alkoxides, citric acid, hydroxides, oxalates, tartaric acid, or other sol–gel procedures to maintain particle dimensions at the nanometer scale from a single-source precursor.

A single-source precursor is a homogeneous, single-phase, amorphous material containing all requisite cations in stoichiometric proportion [22]. This strategy produces homogeneous, multicomponent ceramics at the atomic level. Over the years, numerous functional ceramics such as PbTiO_3_, Pb(Zr_0.53_Ti_0.47_)O_3_, (Ba,Sr)TiO_3_, Pb(Mg_1/3_Nb_2/3_)_0.7_Ti_0.3_O_3_, and BaTiO_3_ have been synthesized from single-source precursors [23]. Single-source precursors for perovskite ceramics fall into two main categories: organometallic precursors (notably alkoxides) and peroxo-hydroxide (or oxyhydroxide) precursors. Peroxo-hydroxide precursors stand out for BaTiO_3_ synthesis because of their ease of preparation and high reproducibility [24,22].

In this study, the synthesis of nonagglomerated nano-t-BaTiO_3_ was explored by hydrothermally treating the barium-titanium peroxo-hydroxide precursor (Ba_2_Ti_2_O(O_2_)_2_(OH)_6_). This work can be considered an extension of previous works that used barium-titanate-peroxo-hydroxide precursors to synthesize BaTiO_3_ ceramics via molten-salt [25] and hydrothermal methods [24,26]. The focus of the current work was the synthesis of the precursor material under various thermal conditions and the hydrothermal synthesis of the precursor under different basic media to minimize the agglomeration between BaTiO_3_ particles and obtain nanosized tetragonal BaTiO_3_.

Previous studies have shown that increasing the NaOH concentration and hydrothermal temperature enables precise control over BaTiO_3_ particle size [24]. Hence, in this work, the precursor material was subjected to concentrated (10 M) mineral base solutions (NH_4_OH, KOH, and NaOH) at various hydrothermal reaction temperatures (100–200 °C). The effect of reaction temperature on the precursor material was also studied by varying the temperature of the peroxo-hydroxide solution (room temperature, 0 °C, and −10 °C). The obtained materials were analyzed by X-ray fluorescence (XRF), scanning electron microscopy (SEM), X-ray diffraction (XRD), Fourier-transform infrared spectroscopy (FT-IR), Fourier-transform Raman spectroscopy (FT-Raman), and thermogravimetric-differential thermal analysis (TGA-DTA).

## 2. Materials and methods

### 2.1. Synthesis of the barium-titanium-peroxo-hydroxide precursor

The precursor material was prepared using barium chloride dihydrate (BaCl_2_·2H_2_O, ≥99.0%, Merck Millipore, Burlington, MA, USA), titanium(IV) chloride (TiCl_4_, 99.9%, Sigma-Aldrich, St. Louis, MO, USA), hydrogen peroxide (H_2_O_2_, 35%, Merck Millipore), and ammonia solution (NH_4_OH, 30%, Merck Millipore).

**Figure 1:**
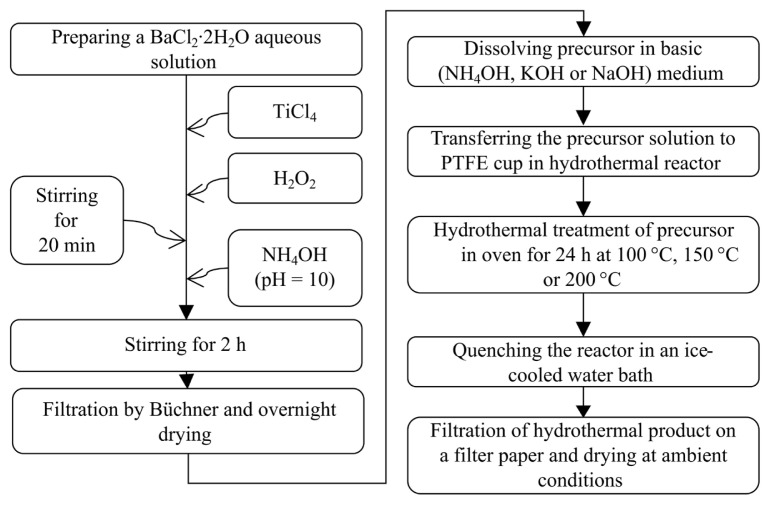
Synthesis procedures for barium-titanium-peroxo-hydroxide (Ba_2_Ti_2_O(O_2_)_2_(OH)_6_) and hydrothermal barium titanate (BaTiO_3_).

The synthesis of the barium-titanium-peroxo-hydroxide precursor is illustrated in Figure 1. In the first stage of the synthesis, a solution with a total concentration of 0.05 M (Ba + Ti), a Ba/Ti molar ratio of 2, a H_2_O_2_/Ti molar ratio of 3, and a total volume of 250 mL was prepared. Initially, BaCl_2_·2H_2_O was dissolved in demineralized water at room temperature. Next, TiCl_4_ was added to the aqueous barium solution under vigorous stirring. After obtaining a clear solution, H_2_O_2_ was added, giving a deep red color due to peroxo complexation, and the mixture was stirred for 20 min. Finally, NH_4_OH was added until the pH reached 10 (pen-type pH meter, HI98100, HI1271, Hanna Instruments, Woonsocket, RI, USA), and the mixture was stirred for 2 h, followed by Büchner filtration under vacuum and drying under ambient conditions for at least 24 h. In addition to the standard procedure described in Figure 1, the solution temperature (room temperature, 0 °C, or −10 °C) and storage periods (1 day or 1 week) of the solutions were varied, as described in Table 1.

### 2.2. Hydrothermal BaTiO_3_ synthesis

Hydrothermal reactions were conducted in a stainless-steel reactor according to the synthesis parameters listed in Table 2. The barium-titanium precursor was hydrothermally treated in NH_4_OH, KOH, or NaOH solution with a concentration of 10 M unless otherwise stated. The high-pressure reactor was then heated at varying temperatures from 100 °C to 200 °C for 24 h. The reactor was cooled in an ice-cooled water bath. The hydrothermal product was washed with demineralized water and dried at room temperature overnight.

### 2.3. Instrumental

Scanning electron microscopy (SEM) images were obtained on a Gemini 300 device (Zeiss, Oberkochen, Germany) with acceleration voltage of 10 keV. Before SEM, the samples were gold-coated to increase their surface conductivity for better image quality. Particle-size distribution histograms were prepared using open-source ImageJ 1.54 software based on a minimum of 100 particles and a maximum of 500 particles observed in the SEM images. Particle lengths were determined from SEM images by measuring nonagglomerated single particles; large particles identified as agglomerates of multiple individuals were excluded to avoid bias in the distribution. Histograms of particle-size distribution were constructed by plotting the relative frequency (%) of particles against particle size (nm), with the x-axis values denoting size intervals between successive tick marks.

XRD diffractograms were collected using an AXS/Discovery D8 diffractometer (Bruker, Billerica, MA, USA) with Cu-Kα radiation (λ = 0.15418 nm). The tube voltage and current were set to 40 kV and 40 mA, respectively. Data were collected over a 2θ range of 5° to 100° with scan step size of 0.02° 2θ and a 0.5-s duration per step. All XRD diffractograms were normalized and analyzed using QualX2 software [27]. Powder XRD data were analyzed by Rietveld refinement using Profex, a graphical user interface for the BGMN refinement engine [28]. Profex provides direct access to BGMN control, structure, and instrument files. The BGMN refinement kernel applies a fundamental-parameters approach, in which the diffraction profile is described as a convolution of wavelength-dependent, instrument-related, and sample-related contributions. Instrumental peak broadening was defined by a precalculated instrument resolution function and was kept fixed during refinement, while only sample-related parameters such as phase scale factors, lattice parameters, microstrain, and crystallite shape were refined. Refinements were performed sequentially, starting with basic parameters and progressively activating anisotropic microstrain, crystallite shape, and lattice parameter models to ensure numerical stability and avoid overparameterization. Background intensities were modeled using polynomial functions and quantitative phase analysis was conducted through the refinement of normalized scale factors. Refinement quality was assessed using standard statistical R-factors, specifically the weighted profile R-factor (R_wp_), the expected statistical R-factor (R_exp_), and goodness-of-fit (GoF), expressed as (χ^2^)^1/2^ with χ^2^ = (R_wp_/R_exp_)^2^, together with visual inspection of the observed, calculated, and difference profiles.

The elemental composition of the samples was analyzed using a supermini200 XRF spectrometer (Rigaku, Tokyo, Japan).

FT-IR measurements were performed on a Spectrum Two ATR FT-IR device (PerkinElmer, Waltham, MA, USA) between 400 and 4000 cm^−1^ with resolution of 1 cm^−1^, 4 scans, and automatic CO_2_ reduction. All FT-IR spectra were normalized.

FT-Raman measurements were performed using an inVia confocal Raman microscope (Renishaw, Wotton-under-Edge, UK) with a minimum step size of 10 nm, repeatability of 1 μm, and 100-nm resolution. A 532-nm (25-mW) laser was used for all measurements. All FT-Raman spectra were normalized.

TGA-DTA measurements were performed using a STAP1600 device (Linseis, Selb, Germany) between 30 °C and 1000 °C in an oxygen atmosphere with a ramp of 5 °C min^−1^. Heat flux was measured as the temperature difference given in μV between the programmed and sample temperatures and was simultaneously measured alongside sample weight loss (%).

## 3. Results and discussion

Elemental analyses of the precursor materials were conducted using XRF. These analyses were used to evaluate the purity of the prepared compounds, the molar ratio of Ba/Ti, and the stoichiometry of Ba, Ti, and oxygen (O). According to the theoretical formula of the precursor, Ba_2_Ti_2_O_5_(OH)_6_, the Ba/Ti molar ratio should be unity. Because the XRF system does not directly measure oxygen, the oxygen stoichiometry was inferred from the oxide forms of the elements.

The properties of the barium-titanium-peroxo-hydroxide precursor have been previously characterized in detail [22]. The present study extends that work by synthesizing the precursor under various temperature conditions, including room temperature, 0 °C, and −10 °C. Furthermore, following the addition of H_2_O_2_ and prior to the introduction of the precipitating agent, the precursor solution was stored at 4 °C for 1 week to evaluate its stability and impact on the final precursor product. The resulting precursor products were analyzed by XRF to assess the barium-to-titanium ratio, which is a good indicator of obtaining the right precursor with the correct stoichiometry. Table 3 presents the weight percentages and mole ratios of barium (Ba) and titanium (Ti) in their oxide forms and other minor elements. The precursor synthesized via the standard procedure exhibited a Ba/Ti molar ratio of 0.73 (ABT-1). Excess Ti was thought to be caused by extensive washing with demineralized water, which resulted in the leaching of Ba^2+^ ions and a lower Ba content than expected. Two precursor solutions (after the addition of H_2_O_2_ and before the addition of NH_4_OH) were prepared and stored for 1 day and 1 week, respectively. After their respective waiting periods, synthesis was resumed by adding the precipitation agent according to the standard procedure. Ba/Ti ratios of 0.66 and 0.82 were found for the solutions stored for 1 day and 1 week, respectively. Compared to the standard procedure, storing the solution yielded a similar (±0.07) Ba/Ti ratio, indicating that the barium-titanium-H_2_O_2_ solution can be stored for extended periods without affecting the final product. The precursor solution was also cooled during synthesis to slow the polymerization reaction of Ti-O-Ti, especially after the addition of the precipitating agent [24,22]. The solution cooled to 0 °C exhibited a Ba/Ti ratio of 0.85. A more significant difference was observed for the solution cooled to −10 °C, which resulted in a Ba/Ti ratio close to unity (1.05), as expected for an ideal BaTiO_3_ precursor. Subsequent hydrothermal reactions were conducted using the precursor prepared at −10 °C. Notably, precipitates from solutions synthesized below room temperature clogged the analytical-grade filter paper (slow filtration), more so for the precursor synthesized at −10 °C. This was believed to be an indication of smaller particle sizes of the precursor material due to a more inhibited Ti-O-Ti polymerization reaction initiated by the addition of the precipitating agent (NH_4_OH).

**Figure 2:**
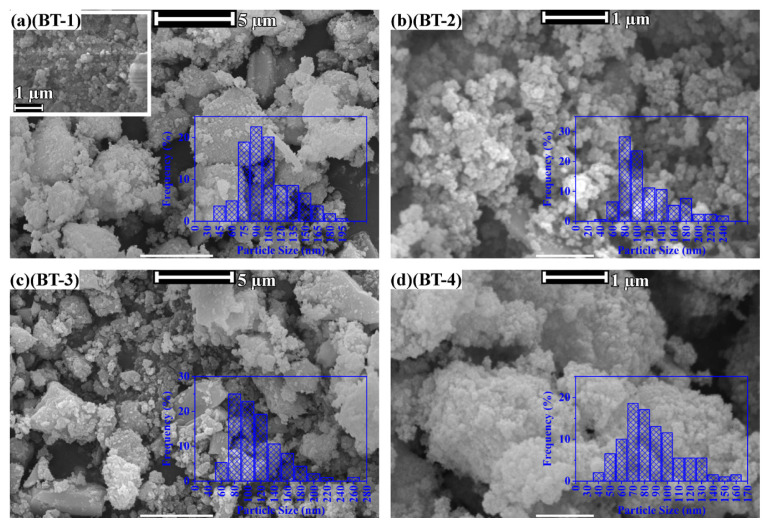
SEM images and particle-size distribution histograms (insets) of hydrothermally produced BaTiO_3_: (**a**, BT-1) 10 M NH_4_OH at 100 °C, (**b**, BT-2) 10 M NH_4_OH at 150 °C, (**c**, BT-3) 5 M NH_4_OH at 100 °C, and (**d**, BT-4) 0.5 M NH_4_OH at 150 °C.

**Figure 3:**
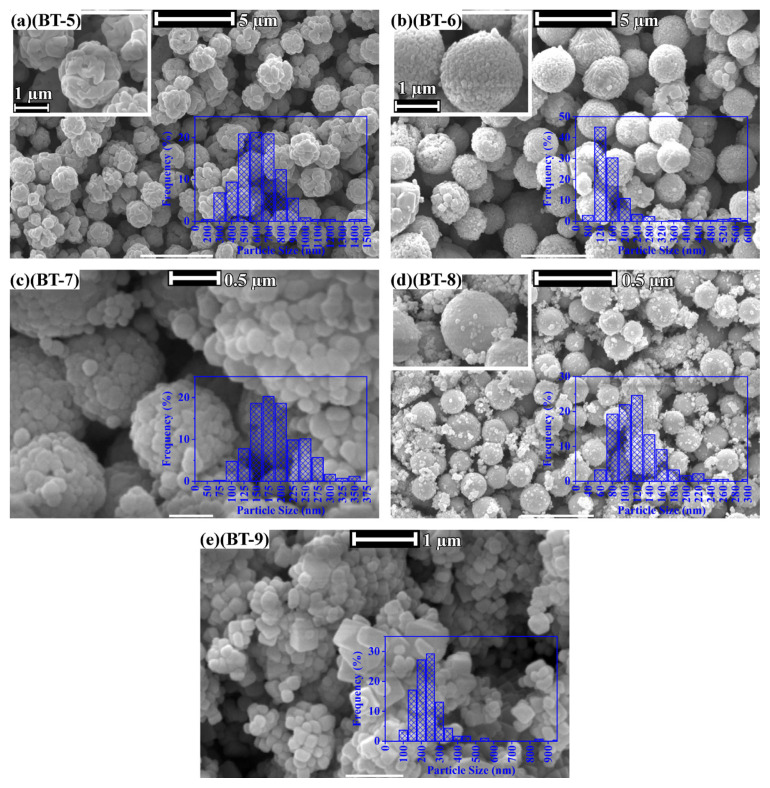
SEM images and particle-size distribution histograms (insets) of hydrothermally produced BaTiO_3_: (**a**, BT-5) 1 M KOH at 150 °C, (**b**, BT-6) 2 M KOH at 150 °C, (**c**, BT-7) 10 M KOH at 150 °C, (**d**, BT-8) 10 M KOH at 100 °C, and (**e**, BT-9) 10 M KOH at 200 °C.

**Figure 4:**
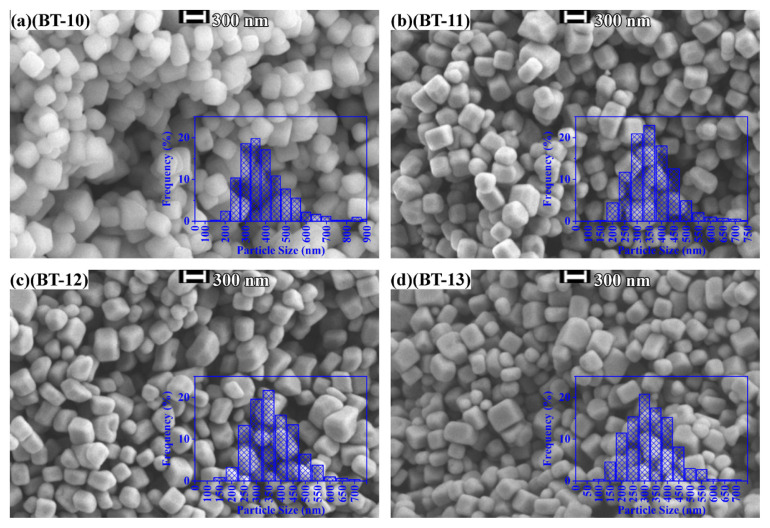
SEM images and particle-size distribution histograms (insets) of hydrothermally produced BaTiO_3_: (**a**, BT-10) 10 M NaOH at 200 °C, (**b**, BT-11) 10 M NaOH at 200 °C in 10 mL of solution, (**c**, BT-12) 10 M NaOH at 200 °C and naturally cooled to room temperature, and (**d**, BT-13) 10 M NaOH at 200 °C with 0.30 M concentration and 10 mL of solution.

Figures 2–4 present SEM images of the syntheses given in Table 2.

SEM images of the hydrothermally treated barium-titanium-peroxo-hydroxide precursors in the NH_4_OH basic environment are shown in Figure 2. The particle-size histograms show that the average nanoparticle size varied around 80 nm regardless of the synthesis parameter. In this study, the term “nanoparticle” refers to nanoscale-derived functional behavior rather than strict ISO size limits. Hence, all particles below 1 μm were referred to as nanoparticles. Independent of concentration and hydrothermal reaction temperature, the NH_4_OH-treated precursor resulted in highly agglomerated particles with irregular, conglomerated larger particles. The hydroxide nature of the basic medium (NH_4_OH) is thought to offer “H-bridges” by adding −OH groups to the surface of BaTiO_3_. Here, “H-bridges” are the hydrogen bond-mediated interactions between the surface hydroxyl (−OH) groups on neighboring BaTiO_3_ particles. Such interactions can promote particle–particle adhesion and lead to agglomeration in strongly alkaline media.

Although the FT-IR spectra of the NH_4_OH-treated samples show vibrational bands typical of BaTiO_3_ (not shown for clarity), the SEM images clearly do not reveal distinct particles with clear particle size and morphologies.

Figures 3a–3e show SEM images of the hydrothermally treated barium-titanium-peroxo-hydroxide precursors in the KOH basic environment. The agglomeration of the particles was more pronounced at a reaction temperature of 100 °C (Figure 3d). Higher reaction temperatures aided the deagglomeration of the precipitated particles, but only to a certain degree (Figure 3e). Precursors treated at a hydrothermal temperature of 150 °C with KOH concentrations of 1, 2, and 10 M (see Figures 3a–3c) showed decreasing average nanoparticle sizes (from ~600 nm to ~175 nm) with increasing KOH concentration, whereas an increase in the average nanoparticle size was observed with increasing hydrothermal temperature (~120 nm to ~250 nm).

The SEM images of the hydrothermally treated barium-titanium-peroxo-hydroxide precursors (quenched in a water bath) in a 10 M NaOH environment at 200 °C revealed cube-like morphologies (Figure 4a). Although some agglomeration remained, it was substantially lower than that observed for BaTiO_3_ samples synthesized in NH_4_OH (Figure 2) and KOH (Figure 3). Decreasing the solution volume from 60 to 10 mL produced sharper cubic particles (Figure 4b), likely driven by a more efficient dissolution–recrystallization cycle during the hydrothermal process. At higher volumes, limited evaporation above the liquid interface hindered this cycle, resulting in less well-defined particles. Similarly, the natural cooling of the reactor following the hydrothermal reaction yielded well-defined cubes with minimal agglomeration (Figure 4c). Reducing the volume to 10 mL and simultaneously increasing the total Ba and Ti concentration to 0.30 M preserved the cube-like morphology but introduced a bimodal particle-size distribution (Figure 4d). Crystals competed for growth space at higher precursor concentrations, triggering a Brownian-type ripening process where larger particles grew at the expense of smaller ones. The average particle size of the individual particles varied between 300 and 350 nm, independent of the synthesis parameters.

The agglomeration behavior of BaTiO_3_ synthesized in different alkaline media is governed primarily by chemical surface interactions and growth kinetics rather than by electrostatic stabilization. Under highly concentrated alkaline conditions (up to 10 M), the electrical double layer surrounding the BaTiO_3_ particles is strongly compressed, rendering classical zeta potential-based descriptions of colloidal stability inapplicable [29,30]. In NH_4_OH and KOH media, extensive surface hydroxylation promoted hydrogen bond-mediated interparticle bridging, leading to the formation of strongly agglomerated clusters, as directly evidenced by SEM observations. In contrast, synthesis in highly concentrated NaOH resulted in markedly reduced agglomeration and well-faceted cubic particle formation. This behavior was consistent with a growth regime dominated by partial surface dissolution followed by recrystallization, which continuously renewed particle surfaces and suppressed persistent hydroxyl-mediated bridging. Consequently, the observed morphology evolution reflected differences in surface chemistry and dissolution–reprecipitation kinetics induced by the alkaline medium rather than surface charge effects, in agreement with previous reports on oxide crystallization under extreme alkaline hydrothermal conditions [24,31,26]. Specifically, in the case of NaOH, the relatively lower apparent agglomeration may be attributed to extreme alkalinity and Na^+^-mediated dissolution kinetics.

These observations underscore the ability of NaOH-mediated hydrothermal synthesis to tune BaTiO_3_ nanoparticle shape and size. Accordingly, all subsequent discussions focus exclusively on NaOH-treated samples and their FT-IR, XRD, FT-Raman, and TGA-DTA analyses.

**Figure 5:**
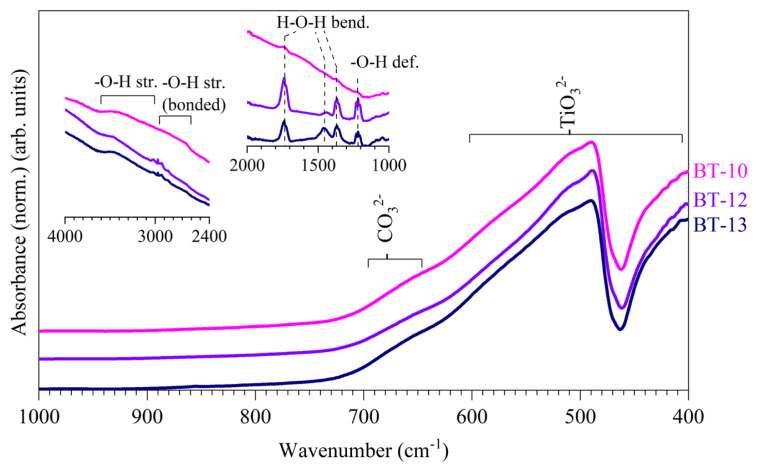
FT-IR spectra of hydrothermally produced BaTiO_3_ acquired at room temperature: (BT-10) 10 M NaOH at 200 °C, (BT-12) 10 M NaOH at 200 °C and naturally cooled to room temperature, and (BT-13) 10 M NaOH at 200 °C with 0.30 M concentration and 10 of mL solution, with insets focused on the regions of 1000–2000 cm^−1^ and 2400–4000 cm^−1^ to highlight lattice water and hydroxyl (−OH) vibrational modes.

The FT-IR spectra (Figure 5) showed vibrational absorbance bands at 410 cm^−1^, 490 cm^−1^, and 570 cm^−1^ typical for titanate (TiO_3_^2−^) modes [32]. A band at approximately 660 cm^−1^ corresponded to carbonate (CO_3_^2−^), resulting from the reaction of Ba^2+^ with CO_2_ species in water and/or BaCO_3_ impurities in the barium precursor [26,32,22]. The insets in Figure 5 focus on the regions of 1000–2000 cm^−1^ and 2400–4000 cm^−1^ to highlight the -O-H vibrational modes. Aside from lattice H_2_O vibrational modes, possible surface -O-H stretching modes (2500–3000 cm^−1^) [32] and -O-H deformation modes (~1220 cm^−1^) [32] are highlighted in the FT-IR spectra. These -O-H vibrational modes are especially relevant in the interparticle H-bridging and, hence, the agglomeration of BaTiO_3_ particles as discussed earlier. Hydroxyl group effects can also be assessed using thermogravimetric analyses such as TGA-DTA, as will be discussed further on.

**Figure 6:**
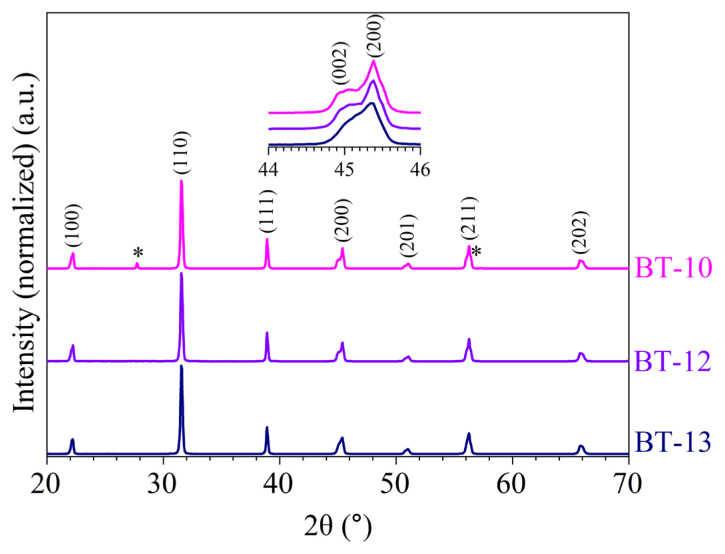
XRD diffractograms of hydrothermally produced BaTiO_3_ acquired at room temperature: (BT-10) 10 M NaOH at 200 °C, (BT-12) 10 M NaOH at 200 °C and naturally cooled to room temperature, and (BT-13) 10 M NaOH at 200 °C with 0.30 M concentration and 10 mL of solution.

XRD diffractograms of samples BT-10, BT-12, and BT-13 obtained in a 10 M NaOH medium are presented in Figure 6. The Miller indices depicted in Figure 6 are the diffraction lines of tetragonal BaTiO_3_ (JCPDS 05-0626). Miller indices for tetragonal BaTiO_3_ are given only on the top curve for the sake of clarity. All XRD diffractograms conform to phase-pure BaTiO_3_ (JCPDS 05-0626). The diffraction lines at approximately 27° and 57.26° (designated in the figure with an asterisk) with minor peaks at 23.9°, 24.2°, 34.0°, and 46.9° (BT-10) corresponded to witherite barium carbonate (BaCO_3_, ICDD 01-071-4900). Because BaCO_3_ is known to accompany barium titanate (BaTiO_3_), the lines at 23.9°, 24.2°, 27.74°, 34.0°, 46.9°, and 57.26° were assigned to BaCO_3_ with very high certainty [24,22,26]. The splitting at ~45° 2θ in the diffractograms (see inset in Figure 6) showed the tetragonality of BaTiO_3_. The greater the splitting, the higher the degree of tetragonality. Sample BT-10 (0.05 M, 60 mL) obtained from the water bath-cooled reactor was relatively more tetragonal and exhibited more tetragonal characteristics due to a larger splitting at ~45° 2θ compared to the sample prepared at a higher concentration (0.30 M) in a smaller volume (10 mL), which exhibited limited splitting of the (002) and (200) lines (BT-13). In the latter, particles were constrained to grow in a relatively smaller space at higher concentrations and smaller solution volumes, resulting in bimodal particles, where the larger particles were tetragonal [25] and the smaller particles were cubic [33]. Natural cooling in the oven (BT-12) resulted in a more pronounced tetragonal crystal phase. This natural, slow cooling resulted in the gradual and controlled transition of the crystal phase from cubic at higher temperatures to tetragonal at room temperature. The similar crystal phase behaviors observed for BT-10 (quenched) and BT-12 (naturally cooled) may have arisen from insufficient cooling efficiency of the water bath, such that the NaOH solution effectively cooled in a quasi-natural manner in both cases. However, rapid quenching in BT-10 likely preserved the witherite BaCO_3_ phase by kinetically arresting its transformation.

**Figure 7:**
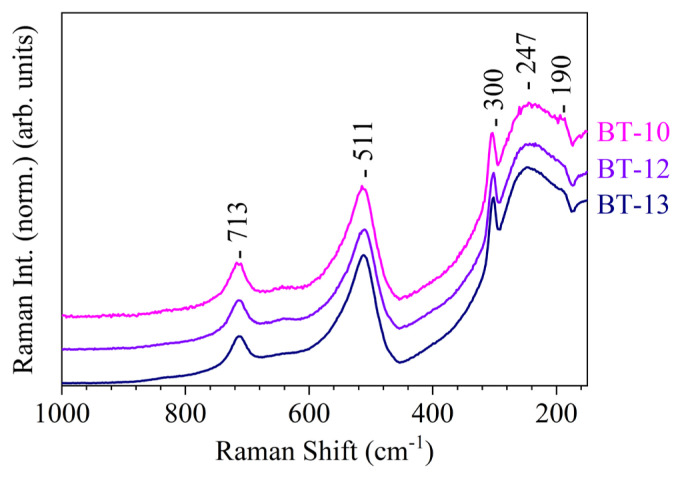
FT-Raman spectra of hydrothermally produced BaTiO_3_ acquired at room temperature: (BT-10) 10 M NaOH at 200 °C, (BT-12) 10 M NaOH at 200 °C and naturally cooled to room temperature, and (BT-13) 10 M NaOH at 200 °C with 0.30 M concentration and 10 mL of solution.

Another technique that can be used to assess a material’s crystalline phase is FT-Raman spectroscopy. In this study, all FT-Raman spectra (Figure 7) showed very clear vibrational modes for BaTiO_3_ at 190 cm^−1^, 247 cm^−1^, 300 cm^−1^, 511 cm^−1^, and 714 cm^−1^ [24,32]. Because Raman-active modes arise only in noncentrosymmetric structures, the presence of these bands confirmed the existence of tetragonal BaTiO_3_ domains in all samples. While the XRD diffractograms clearly revealed variations in crystal symmetry as a function of synthesis parameters, most notably through changes in the (002)/(200) peak splitting, no pronounced differences were observed among the FT-Raman spectra. This behavior was attributed to the use of a 532-nm excitation laser, which significantly enhances Raman scattering efficiency and renders the technique highly sensitive to even small fractions of tetragonal domains. Consequently, FT-Raman spectroscopy confirmed the presence of tetragonal BaTiO_3_ but was less sensitive to subtle variations in tetragonality or relative phase fractions than XRD. These results highlight the complementary nature of XRD and Raman spectroscopy, as XRD provided quantitative phase and symmetry information and Raman spectroscopy selectively probed local noncentrosymmetric distortions.

The FT-Raman spectra in Figure 7 and the powder XRD diffractograms in Figure 6 qualitatively illustrate the crystalline phases and the peak splitting associated with tetragonality. Quantitative phase fractions and c/a ratios were obtained by Rietveld refinement.

**Figure 8:**
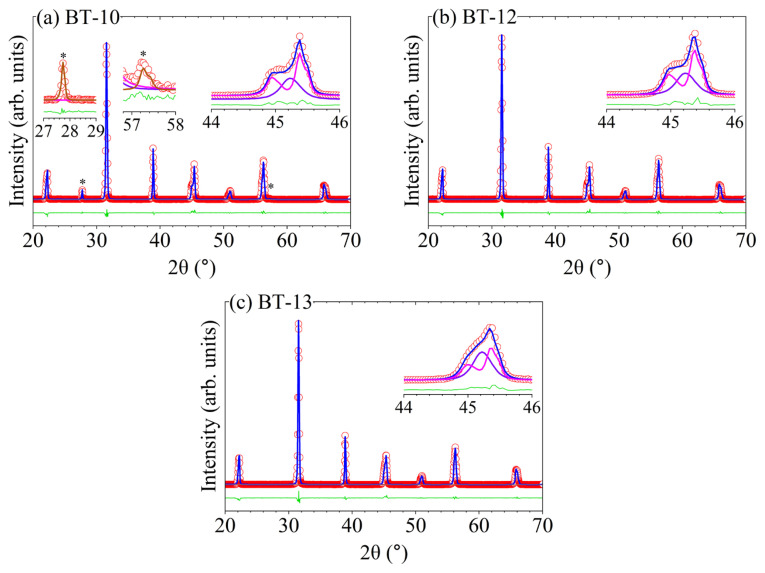
Rietveld fit for powder XRD data acquired at ambient temperature with λ = 0.15418 nm: (**a**, BT-10) 10 M NaOH at 200 °C, (**b**, BT-12) 10 M NaOH at 200 °C and naturally cooled to room temperature, and (**c**, BT-13) 10 M NaOH at 200 °C with 0.30 M concentration and 10 mL of solution. The red circles, blue lines, and green lines represent the observed, calculated, and difference profiles, respectively. The violet, magenta, and brown lines represent cubic BaTiO_3_, tetragonal BaTiO_3_, and witherite BaCO_3_ phases, respectively. Insets for all plots focus on the region of 44° to 46° 2θ to highlight the (002)/(200) tetragonal phase splitting. Additional insets for BT-10 focus on 2θ regions 27° to 29° and 57° to 58° to highlight the witherite BaCO_3_ phase (*).

Rietveld refinement was used to analyze the diffraction data of samples BT-10, BT-12, and BT-13 to quantify the relative phase fractions and extract precise lattice parameters for the coexisting BaTiO_3_ polymorphs (Figure 8). All refinements converged reliably with low residuals (R_wp_ ≈ 8%, χ^2^ ≈ 1.6), confirming an excellent agreement between the observed and calculated diffraction profiles (Table 4). Anisotropic microstrain, crystallite shape, and lattice parameters were refined for both phases to accurately describe the diffraction profiles and avoid artificial peak broadening. The absence of systematic features in the difference plots confirms that the applied two-phase model adequately captures the sample’s structural complexity without overparameterization.

The refinements confirmed the coexistence of cubic and tetragonal BaTiO_3_ phases in all samples, in agreement with the aforementioned peak splitting behavior in Figure 6. Quantitative phase analysis revealed a systematic variation in phase fractions as a function of the synthesis conditions. Sample BT-10 contained a higher proportion of the tetragonal phase (57.1 wt.%) relative to the cubic phase (42.9 wt.%), whereas BT-12 exhibited a decrease in tetragonal content (56.3 wt.%). BT-13 exhibited a nearly equimolar distribution of cubic (53.4 wt.%) and tetragonal (46.6 wt.%) BaTiO_3_. The estimated standard deviations of approximately ±2 wt.% indicated that the phase fractions were statistically significant (Table 4).

These results quantitatively support the qualitative XRD observations, where enhanced tetragonality was associated with BT-10. The stabilization of a substantial cubic fraction at room temperature, particularly in BT-13, is consistent with nanoscale effects, where particle size, internal strain (e.g., due to hydroxyl defects and oxygen vacancies [34–36]), and surface energy contributions suppress the complete cubic-to-tetragonal transformation typically observed in bulk BaTiO_3_ [31].

The refined lattice parameters further supported this interpretation. The cubic BaTiO_3_ phase exhibited lattice parameters in the range of a = 0.400999–0.401074 nm that was slightly expanded relative to bulk values, which can be attributed to residual lattice strain and size-related effects inherent to hydrothermally synthesized nanoparticles [21]. For the tetragonal phase, the refined lattice parameters (a ≈ 0.3996154–0.399741 nm, c ≈ 0.402877–0.403310 nm) yielded c/a ratios of approximately 1.007–1.009, which was characteristic of room-temperature tetragonal BaTiO_3_. Notably, the degree of tetragonality increased from BT-13 to BT-10, in line with the increasing prominence of tetragonal peak splitting observed in the XRD patterns (Figure 6) [37]. A minor witherite BaCO_3_ phase was also detected in BT-10, consistent with the weak carbonate reflections observed in the XRD patterns (Figure 6) [38].

The combined XRD and SEM results revealed a clear structure–morphology relationship in the hydrothermally synthesized BaTiO_3_ samples. Rietveld refinement confirmed that samples exhibiting higher tetragonal phase fractions corresponded to the larger, well-faceted cubic particles observed by SEM (e.g., BT-10), whereas samples with increased cubic phase content displayed smaller particle sizes and weaker tetragonal distortion (e.g., BT-13). This behavior was consistent with size-dependent phase stability in BaTiO_3_, where reduced particle dimensions and increased surface energy suppressed the complete cubic-to-tetragonal transformation at room temperature [9]. In samples prepared at a higher precursor concentration and reduced solution volume (BT-13), SEM revealed a bimodal particle-size distribution, which correlated with the nearly equimolar cubic–tetragonal phase fractions obtained from Rietveld refinement. These observations indicated that larger particles preferentially stabilized the tetragonal phase, whereas smaller particles retained cubic symmetry, providing direct experimental evidence for a size-mediated structure–morphology relationship [33,9,31].

**Figure 9:**
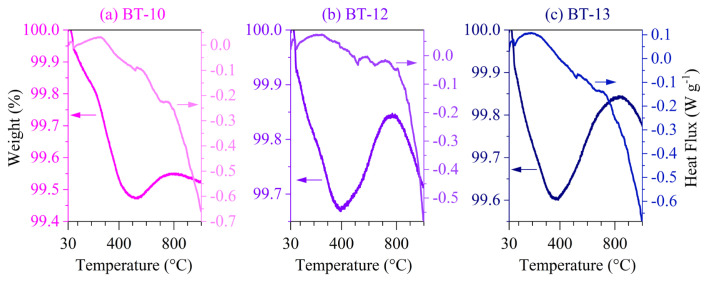
TGA-DTA performed under dry atmosphere and 5 °C min^−1^ heating rate for hydrothermally produced BaTiO_3_: (**a**, BT-10) 10 M NaOH at 200 °C, (**b**, BT-12) 10 M NaOH at 200 °C and naturally cooled to room temperature, and (**c**, BT-13) 10 M NaOH at 200 °C with 0.30 M concentration and 10 mL of solution.

TGA-DTA thermograms obtained under dry atmosphere for samples BT-10, BT-12, and BT-13 are shown in Figure 9. The TGA-DTA curves were typical for phase-pure BaTiO_3_ samples with minimal BaCO_3_ contamination [22].

The TGA curves in Figure 9 show a total weight loss of approximately 0.5% between 30 °C and 430 °C. The temperature range from 30 °C to 230 °C corresponded to the dehydration of surface-adsorbed water molecules, as also observed by FT-IR spectroscopy (Figure 5), and coincided with an endothermic heat flux [22]. A second weight-loss step, observed between 230 °C and 400 °C, was attributed to the dehydroxylation of surface −OH groups (see also Figure 5), again accompanied by an endothermic heat flux [22]. The weight loss occurring between 800 °C and 1000 °C was assigned to the decomposition of BaCO_3_ [39].

A small but progressively increasing weight gain was observed between 400 °C and 800 °C. This weight gain was attributed to oxygen uptake associated with oxygen vacancies and hydroxyl-related lattice defects, which are commonly reported for hydrothermally synthesized BaTiO_3_ [34–36]. The highest weight increase was observed for BT-13 (0.24%), which was hydrothermally prepared using a lower solution volume (10 mL) and a higher precursor concentration (0.30 M). This behavior can be rationalized by considering that BaTiO_3_ nuclei formed under constrained solution volumes experience enhanced defect formation, leading to metastable cubic BaTiO_3_ phases [40], as evidenced by the XRD patterns in Figure 6 and 8. Increasing the solution volume to 60 mL for sample BT-12 resulted in a comparatively lower weight gain (0.18%), which was attributed to hydroxyl groups incorporated into the BaTiO_3_ lattice during the slow cooling process. The relatively high O–H vibrational intensities observed in Figure 5 for both BT-13 and BT-12 supported the presence of such hydroxyl-related lattice defects. In contrast, the water bath-quenched sample BT-10 exhibited lower weight gain, consistent with reduced interaction with the hydroxyl-rich environment. This behavior correlated with a higher degree of tetragonality, as indicated by the pronounced splitting of the XRD reflection at ~45° 2θ in Figures 6 and 8. The comparatively low O–H vibrational intensities observed for BT-10 in Figure 5 further supported this interpretation.

## 4. Conclusion

This study demonstrates that both the precursor chemistry and the hydrothermal alkaline environment play decisive roles in controlling the agglomeration, morphology, and crystal phase of BaTiO_3_ nanoparticles. The barium-titanium peroxo-hydroxide precursor can be stored for extended periods without degradation; however, synthesis at subzero temperatures (−10 °C) effectively suppresses Ti-O-Ti polymerization, yielding near-stoichiometric precursors with reduced particle size. These precursors provide a favorable starting point for hydrothermal BaTiO_3_ formation.

Hydrothermal treatment in NH_4_OH and KOH results in severe particle agglomeration, which is attributed to extensive surface hydroxylation and hydrogen bond-mediated interparticle interactions. Increasing the KOH concentration and reaction temperature partially reduces agglomeration but does not prevent the formation of large conglomerates. In contrast, hydrothermal processing in highly concentrated NaOH (10 M) at 200 °C produces well-defined, weakly agglomerated cubic BaTiO_3_ particles. This behavior is attributed to a dissolution–recrystallization growth regime under extreme alkalinity, specifically in a Na^+^-environment, which continuously renews particle surfaces and suppresses hydroxyl-mediated bridging.

Quantitative Rietveld refinement confirmed that synthesis parameters such as cooling rate, solution volume, and precursor concentration govern the balance between cubic and tetragonal BaTiO_3_ phases through size- and strain-dependent stabilization mechanisms. Overall, the combination of low-temperature, single-source precursor synthesis and NaOH-mediated (mainly by means of Na^+^ ions) hydrothermal processing offers a robust and scalable route to morphology-controlled BaTiO_3_ nanoparticles with minimized agglomeration.

## Figures and Tables

**Table 1:** Parameters varied during the synthesis of the barium-titanium-peroxo-hydroxide (Ba_2_Ti_2_O(O_2_)_2_(OH)_6_).

Sample	Solution temperature*	Stirring after the addition of H_2_O_2_	Storage period after the addition of H_2_O_2_ and before the addition of NH_4_OH
ABT-1	RT**	20 min	-
ABT-2	RT	20 min	1 day at 4 °C
ABT-3	RT	20 min	1 week at 4 °C
ABT-4	0 °C	20 min	-
ABT-5	−10 °C	20 min	-

* Measured using a glass thermometer.

** RT: Room temperature.

**Table 2:** Synthesis parameters for the hydrothermal preparation of BaTiO_3_.

Sample	Reactor temperature (°C)	Solution volume (mL)	Reactor cooling	Total Ba+Ti (M)	NaOH (M)	KOH (M)	NH_4_OH (M)
BT-1	100	60	water bath*	0.05	-	-	10
BT-2	150	60	water bath	0.05	-	-	10
BT-3	100	60	water bath	0.05	-	-	5
BT-4	150	60	water bath	0.05	-	-	0.5
BT-5	150	60	water bath	0.05	-	1	-
BT-6	150	60	water bath	0.05	-	2	-
BT-7	150	60	water bath	0.05	-	10	-
BT-8	100	60	water bath	0.05	-	10	-
BT-9	200	60	water bath	0.05	-	10	-
BT-10	200	60	water bath	0.05	10	-	-
BT-11	200	10	water bath	0.05	10	-	-
BT-12	200	60	natural cooling	0.05	10	-	-
BT-13	200	10	water bath	0.30	10	-	-

* Ice-cooled water bath.

**Table 3:** X-ray fluorescence (XRF) analysis results for the barium-titanium-peroxo-hydroxide precursors synthesized according to Table 1.

Sample		BaO	TiO_2_	Cl	K_2_O	NiO	Ag_2_O	O	Ba/Ti
ABT-1	Mol%	16.3	22.2	0.6	-	-	-	60.8	
	Stoichiometry	1.00	1.36					3.72	0.73
ABT-2	Mol%	15.0	22.7	1.5	0.0	0.1	0.0	60.6	
	Stoichiometry	1.00	1.51					4.04	0.66
ABT-3	Mol%	17.4	21.2	1.5	0.1	0.0	0.0	59.8	
	Stoichiometry	1.00	1.22					3.44	0.82
ABT-4	Mol%	17.7	20.9	1.8	0.1	-	-	59.6	
	Stoichiometry	1.00	1.18					3.37	0.85
ABT-5	Mol%	20.0	19.1	2.3	0.1	-	-	58.4	
	Stoichiometry	1.05	1.00					3.05	1.05

**Table 4:** Rietveld fitting results with R_wp_ (weighted profile R-factor), R_exp_ (expected statistical R-factor), the agreement factor (χ^2^), and goodness-of-fit (GoF) parameters along with compound weights, R_phase_, and lattice parameters for samples: (BT-10) 10 M NaOH at 200 °C, (BT-12) 10 M NaOH at 200 °C and naturally cooled to room temperature, and (BT-13) 10 M NaOH at 200 °C with 0.30 M concentration and 10 mL of solution.

Refinement fits	BT-13		BT-12		BT-10	
R_wp_ (%)	7.94		8.01		7.95	
R_exp_ (%)	6.24		6.21		6.17	
χ^2^	1.6191		1.6637		1.6602	
GoF	1.2724		1.2899		1.2885	
Backgr.Coeff.	10		10		10	
Iterations	10		10		10	
***BaTiO*** *_3_* *** cubic (Pm-3m / no:221)***						
Comp.(wt%)	53.4	± 2.0	43.7	± 1.6	33.1 (42.9)*	± 4.5
R_phase_ (%)	5.26		5.33		5.46	
a (nm)	0.401074	± 0.000016	0.400999	± 0.000017	0.401019	± 0.000015
***BaTiO*** *_3_* *** tetragonal (P4mm / no:99)***						
Comp.(wt%)	46.6	± 2.0	56.3	± 1.6	44.1 (57.1)*	± 5.9
R_phase_ (%)	6.04		6.01		6.25	
a (nm)	0.399741	± 0.000010	0.3996579	± 0.0000067	0.3996154	± 0.0000060
c (nm)	0.402877	± 0.000022	0.403061	± 0.000014	0.403310	± 0.000011
c/a	1.0078		1.0085		1.0092	
***BaCO*** *_3_* *** witherite (Pnma / no:62)***						
Comp. (wt%)					22.8	± 9.9
R_phase_ (%)					9.60	
a (nm)					0.643299	± 0.000033
b (nm)					0.53208	± 0.00046
c (nm)					0.89036	± 0.00047

* In the case where the witherite BaCO_3_ phase is excluded.
